# A Tutorial on Mechanical Decision-Making for Personnel and Educational Selection

**DOI:** 10.3389/fpsyg.2019.03002

**Published:** 2020-01-23

**Authors:** Rob R. Meijer, Marvin Neumann, Bas T. Hemker, A. Susan M. Niessen

**Affiliations:** ^1^Department of Psychometrics and Statistics, Faculty of Behavioral and Social Sciences, University of Groningen, Groningen, Netherlands; ^2^Department of Psychometrics and Research in Educational Measurement, Cito, Arnhem, Netherlands

**Keywords:** mechanical prediction, clinical prediction, decision-making, educational selection, personnel selection, prediction

## Abstract

In decision-making, it is important not only to use the correct information but also to combine information in an optimal way. There are robust research findings that a mechanical combination of information for personnel and educational selection matches or outperforms a holistic combination of information. However, practitioners and policy makers seldom use mechanical combination for decision-making. One of the important conditions for scientific results to be used in practice and to be part of policy-making is that results are easily accessible. To increase the accessibility of mechanical judgment prediction procedures, we (1) explain in detail how mechanical combination procedures work, (2) provide examples to illustrate these procedures, and (3) discuss some limitations of mechanical decision-making.

*Research does not win victories in the absence of committed policy advocates, savvy political work and happy contingencies of time, place and funds* (Weiss and Bucuvalas, 1980).

*[When it comes to prediction], the whole trick is to decide what variables to look at and then to know how to add* (Dawes and Corrigan, 1974).

Decision-making plays a crucial role in recruitment, selection, and hiring practices for jobs and admission procedures for education. In making such decisions, much effort goes into selecting the right predictors and collecting relevant information. However, the quality of the decisions is dependent not only on the quality of the information but also very much on how the information is combined.

There are robust empirical research findings that show that, when making decisions, it is better to combine information (e.g., test scores, interview scores) according to some decision rule than to combine data intuitively, or “in the mind” (e.g., Meehl, 1954; Grove and Meehl, 1996; Ganzach et al., 2000; Grove et al., 2000; Aegisdóttir et al., 2006; Kuncel et al., 2013). A famous example of a very simple successful mechanical decision rule is the Apgar score [see Kahneman (2011, p. 227), in which a newborn gets a score (0, 1, or 2) on five dimensions: heart rate, respiration, reflex, muscle tone, and color] instead of providing an intuitive judgment of the newborn. This latter form of judgment is often referred to as “holistic” or “clinical” judgment, whereas combining information based on a decision rule is described as “statistical,” “actuarial,” or “mechanical” judgment. Thus, for example, when deciding which candidate to select for a job, better, more predictive judgments are made when taking the sum of the (transformed) scores on, say, an intelligence test and the ratings derived from a structured interview, than when combining these scores in an intuitive way, without using an explicit rule (e.g., Dawes, 1979).

In order to make accurate judgments, decision makers need to use valid predictors and weigh information accurately and consistently across cases (Karelaia and Hogarth, 2008). Many studies have shown that decision makers have much difficulty in consistently weighting information across persons (Yu, 2018). Furthermore, people weigh cues inaccurately (Dawes, 1979; Kausel et al., 2016) and tell themselves, or others, coherent stories based on information that does not have much predictive validity, like impressions from unstructured interviews (Dana et al., 2013). Kahneman and Klein (2009, p. 520) discussed that skilled intuitive decision-making (similar to skilled holistic decision-making, in our terminology) is based on recognition. To develop recognition, we need “high-validity environments” and “an opportunity to learn.” The problem with contexts like personnel selection is that these conditions are not satisfied; even with the best methods, future performance is far from perfectly predictable, and explicit feedback is often absent, delayed, and incomplete.

Thus, although there is overwhelming evidence (e.g., Kuncel et al., 2013) that mechanical judgment, in general, yields equally valid or more valid predictions and better decisions than holistic judgment, decision makers and policy makers seldom use these research findings. Many decision makers embrace holistic judgment (e.g., Ryan and Sackett, 1987; Vrieze and Grove, 2009; Silzer and Jeanneret, 2011). For example, colleges advertise that they weigh factors like hardships and service to the community holistically to get a more accurate and fair impression of a candidate (Hartocollis, 2019).

Highhouse (2008) provided various explanations for the reluctance to use mechanical rules in practice. One explanation is that practitioners lack knowledge on the benefits of mechanical over clinical judgment. Another explanation is that, on an individual level, using mechanical combination procedures may result in a lack of perceived autonomy and social interaction people desire when they discuss candidate information and make decisions. As Swets et al. (2000) discussed for diagnostic decision-making: “[*mechanical methods*] *are often met with resistance, especially if they are seen as replacing or degrading clinicians. Further, diagnosticians want to feel that they understand their own diagnoses and recommendations and that they can give a narrative of their thought processes.*”

The underutilization of mechanical judgment by practitioners and policy makers also illustrates the broader difficulties of making practice and policies evidence-based. That is, to design policies that are based on well-grounded scientific findings. To stimulate evidence-based decision-making, it is important to consider the conditions under which policy makers adopt research findings. The British Academy (2008; cited in French, 2018) discussed that policy makers want research findings that “(1) are relevant (2) timely (3) robust (with methodology uncontested) (4) applicable to the issue of concern (5) are accessible to a wider audience (6) bring together relevant expertise from a number of disciplines (7) have champions and advocates (8) involve the users of research in the research project from the outset (co-production model) (9) support existing ideologies and are uncontentious.”

When we evaluate mechanical decision-making against these criteria, it is clear from the meta-analyses by Grove et al. (2000), Kuncel et al. (2013), and Aegisdóttir et al. (2006) that mechanical prediction is relevant, timely, robust, and applicable to the issue of concern (criteria 1 through 3) and has its advocates and champions in science (criterion 7). Furthermore, it brings together expertise from disciplines like psychology and statistics (criterion 6). Mechanical decision-making is very relevant (criterion 4); however, it is not felt as relevant because many practitioners do not have the impression that their decisions are suboptimal. The challenge is to let the larger audience experience the relevance of mechanical decision-making. Another challenge is the accessibility of these findings to a wider audience (criterion 5). Consequently, the misunderstandings about mechanical decision-making may lead to debates and may promote perceptions of dehumanization (criterion 9). Finally, the lack of involving researchers from the outset in a project (criterion 8) is an issue. Lilienfeld et al. (2013) also discussed that there is a lack of papers that translate “findings into non-technical ‘bottom-line’ conclusions that practitioners can readily digest and use” (p. 897). Vrieze and Grove (2009) conducted a survey under clinical psychologists to investigate how many of them used mechanical decision-making. From the logical inconsistencies in some of their answers, it can be deduced that clinicians had difficulty in understanding what mechanical decision-making exactly is, or how to apply it.

The aim of this paper is to illustrate the suboptimal nature of holistic decision-making (criterion 4) and to increase understanding of mechanical judgment procedures (criterion 5). Therefore, in this paper, we (1) explain in detail how mechanical judgment procedures work, (2) provide simple examples to illustrate how these procedures can be designed, and (3) discuss common objections and overlooked advantages. We also discuss some limitations of mechanical procedures in practice that are not often mentioned in the literature.

We fully recognize that lack of the accessibility of knowledge and the existence of some misunderstanding around mechanical procedures are not the sole explanations for the underutilization of mechanical judgment (we return to this issue in the *Discussion* section below). However, they are crucial for decision makers to apply mechanical judgment procedures, and existing studies do not provide explicit guidelines as detailed as we do in this tutorial. We first discuss mechanical and holistic judgment and then we provide an example how mechanical decision-making can be implemented in selection procedures.

## Mechanical Versus Holistic Judgment

In a seminal book on mechanical versus holistic judgment, Meehl (1954) synthesized research that showed that *combining* information according to a rule instead of intuition results in better predictions and decisions. In holistic judgment, information from different sources is combined (in the mind) to form a hypothesis about a candidate, and then based on this hypothesis, “we arrive at a prediction what is going to happen” (Meehl, 1954, p. 4). Hence, the definition of holistic judgment is not restricted to judgments that are based on “gut feelings” that lack substantiation. Decision makers can and do often provide an explanation of the reasoning behind their holistic judgments. However, a substantiated judgment made “in the mind” is still defined as a holistic judgment.

It is also very important to realize that the difference between holistic and mechanical judgment is concerned with the *combination* of information; it is *not* about what kind of valid information is used. For example, interview scores can be combined with test scores according to some mechanical rule, although these interview scores may contain a subjective element. Experts and specialists remain indispensable, but their primary responsibility is to ensure that we choose *valid* information to base judgments and decisions on Meehl (1956). The choice of what information to include can be based on, for example, knowledge of the scientific literature or valid local research findings. Next, the information is combined on the basis of a rule, *not* on the basis of professional experience, intuition, or a “holistic” combination of information. Such rules can be very complex or very simple, as we will illustrate below.

It appears to be very difficult for professionals to use decision rules without being tempted to add information that is not in the decision rule, or without altering mechanical judgments (Eastwood et al., 2012; Dietvorst et al., 2018). This is probably because in everyday decision-making (e.g., what to have for breakfast), we also combine information and make decisions holistically.

Hence, decision rules lead to better predictions, but for practitioners, it is often not very straightforward how they can be applied. Therefore, below we provide an extensive illustration with examples of different *simple* rules. More complicated rules can be used (for example, one may weigh each predictor with the predictive validity obtained from a meta-analysis, or one may optimize weights that meets organizational objectives, such as hiring a certain number of women in the organization), but here we restrict ourselves to simple rules that can be easily applied in practice and that often perform equally well as more complicated rules (Dawes, 1979; Dana and Dawes, 2004). We provide examples for personnel and educational selection and assessment procedures.

## An Illustration: How to Decide Which Candidate to Select for a Job

**FIGURE 1:**
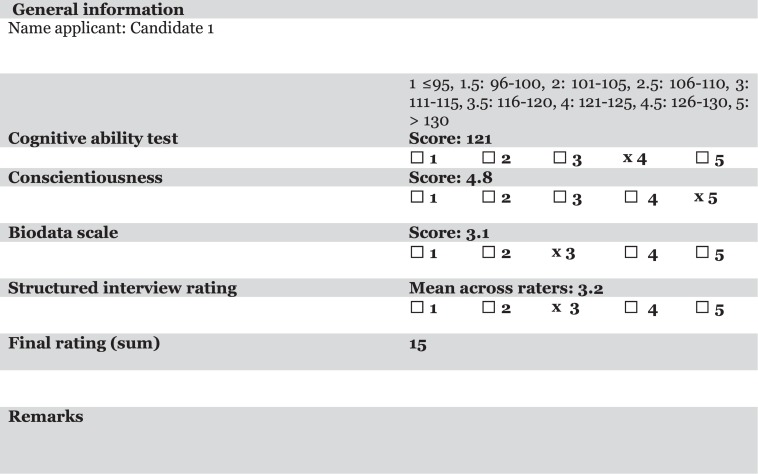
Example of a joint candidate assessment form. The score on the cognitive ability test is rescaled to a score of 4 on a 5-point scale.

We start with illustrating mechanical judgment in the context of personnel selection, that is, the selection of a candidate for a middle-management position. We distinguish different steps in our procedure:

*Step 1. Specify your criteria.* Start with specifying the job performance criteria; that is, what do you want to predict? This may seem obvious, but it is often overlooked as an explicit step. For example, do you want to predict how well candidates will perform their future tasks, or do you also want to select a friendly colleague?
*Step 2. Choose your predictors.* In this step, you decide what information to collect in order to make the desired predictions and how to collect that information. This may, for example, include scores on psychological tests and ratings based on structured interviews. In this step, it is important that the information that is considered is valid. In practice, you may have to rely on results from meta-analyses that discuss how valid psychological tests, structured interviews, or personality questionnaires are in high-stakes selection contexts. However, you may also use local research findings, given that this information is reliable and valid (correct design, adequate sample size). Thus, information should be included based on empirically established relations with criterion scores, like future job performance. Note, again, that a mechanical combination of information (predictors) does not imply that “subjective” impressions cannot be considered: the combination rule is mechanical, not the information collection. However, the information should be quantified.
  In the selection procedure for the middle-management position, we use the score on a cognitive ability test, the score on a conscientiousness scale from a personality inventory, a score from a biodata scale, and a score based on a structured interview^1^.
*Step 3. Collect the information.* This includes, for example, administering tests, conducting interviews, and/or rating resumes. The information is collected without making judgments or decisions other than on the traits, skills, and abilities that are assessed. A structured interview can be scored by multiple interviewers. In that case, the interview ratings are averaged into a final interview rating.
*Step 4. Combine information according to a rule.* We will illustrate this below using a number of alternatives discussed by Yu (2018) and Kuncel (2008): (1) equal weighting, (2) weights obtained via experts, (3) holistic and mechanical synthesis (Sawyer, 1966), and (4) limited expert judgment. These methods are described below. One important condition for combining the information is using the same scale for all traits and skills that are assessed. This may be done through standardizing the scores and ratings. However, if this is not feasible, for example, due to a small number of candidates or lack of a database containing scores and ratings of candidates, a method such as presented in Figure 1 and to be discussed below can be used^2^. Finally, compute the final scores and decide who to select.

## Different Ways to Combine Information

The most important advantage of using a rule is that it will result in consistent weights for the different types of information. Using consistent weights will in most cases result in better decisions than when information is combined holistically (Karelaia and Hogarth, 2008; Yu, 2018). Let us illustrate the rules on the basis of the assessment form depicted in Figure 1.

As discussed above, four types of information were collected: (1) cognitive ability test scores, (2) conscientiousness scale scores, (3) biodata scale scores, and (4) interview scores. To be able to combine these scores in a simple and meaningful way, all predictors were scored on or converted to a scale of 1–5. The general formula to determine a “suitability score” equals:

Suitability score=cognitive ability test score×(w)c⁢a+conscientiousness score×(w)c⁢s+biodata score×(w)b+interview rating×(w)i,

where the *w’s* are the weights that can be given to the different scores and ratings.

When using statistical models such as regression models, these weights are estimated based on a dataset that contains the predictors and a criterion measure, such as job performance. However, in practice, data to estimate optimal regression weights are often not available. Therefore, researchers have investigated whether simple solutions work as well as regression models, or at least better than holistic judgment (Dawes, 1979; Bobko et al., 2007; Kuncel, 2008; Dietvorst et al., 2018; Yu, 2018). The conditions needed for these simple alternatives are that each score or rating is *positively related* to the outcome we want to predict and positively related to each other. We largely followed Yu (2018), who discussed a number of alternatives to regression-based weights when using mechanical rules. The different approaches are ordered from simple to more complex and – roughly – from most to least appropriate; the last two approaches still need some holistic judgment or overruling the results of the mechanical prediction. Such exceptions made by human judges tend to reduce the quality of judgments and predictions (Dawes, 1979). However, in general, all these procedures should lead to better judgments and predictions as compared to a full holistic judgment.

In Table 1, we provide the rescaled scores of five hypothetical candidates on the four predictors and the scores for different scoring rules, if applicable. We use these scores to illustrate the use of the different decision rules.

**TABLE 1:** Scores on different predictors for five candidates.

Assessment scores					Final scores			
Candidate	Cognitive ability test	Conscientiousness	Biodata	Interview	Equal weights	Expert weights	Mechanical synthesis	
							Holistic rating	Total score
1	4	5	3	3	15	22	3	18
2	3.5	2	3.5	4	13	20.5	3.5	16.5
3	2	3.5	2.5	3	11	16	3.5	14.5
4	4	4	3.5	2	13.5	19.5	2	15.5
5	5	5	4	3	17	25	2.5	19.5

### Method 1: Equal Weighting

In this procedure, each predictor score is weighted equally and gets a weight of 1. Thus, each score or rating is considered equally important, and the suitability score (*S*) using this prediction rule for candidate 1 (see Table 1) is, *S* = 4 + 5 + 3 + 3 = 15. This simple rule can be applied for every candidate, and the candidate with the highest mean score across the raters is selected. This method often works almost as well as regression-based weighting, especially when the sample on which regression analysis is conducted is not large (say, not larger than 200) and when the optimal regression-based weights would not differ much from each other (Bobko et al., 2007). However, recently, Sackett et al. (2017) showed that when we apply equal weighting and (1) there is one strong predictor (such as intelligence test scores), (2) the other predictors have a relatively weak relation with the criterion, and (3) the intercorrelations between the predictors are relatively strong, adding a second or third predictor may reduce the predictive validity as compared to using only one best predictor. The reader may consult Sackett et al. (2017), who provide a table with predictive validity coefficients resulting from equally weighting different combinations of information.

### Method 2: Weights Provided by Experts

Decision makers may have reasons to consider particular information about a candidate more important than other information. For example, in our example of selecting a candidate for a middle-management position, we can decide to give more weight to the cognitive ability test score because research has shown that these test scores have, in general, a strong relationship with future job performance (Schmidt and Hunter, 1998). Furthermore, we may give the structured interview rating more weight because we used the interviews to assess valid information about the skills and traits that are very relevant for this particular job. If we weight the cognitive ability test score and the interview rating twice as much as the conscientiousness score and the biodata scale score, our rule equals:

Suitability score=cognitive ability test score×(2)+conscientiousness score×(1)+biodata score×(1)+interview rating×(2).

For candidate 1, we get *S* = 4(×2) + 5 + 3 + 3(×2) = 22. We can also observe in Table 1 that, although the highest scoring candidate is the same as when all predictors were given equal weights, the ordering of the candidates changed. In practice, this approach tends to yield very similar results as when equal weights are used (Bobko et al., 2007).

### Method 3: Mechanical Synthesis

Under this rule, a decision maker first makes a holistic judgment of the suitability of the candidate. However, this holistic judgment is quantified on the same scale as the other information and is made in addition to the other information that is collected.

In the example provided in Figure 1, an additional row would be added for this overall holistic judgment, scored on a scale of 1–5. This holistic rating is then added as an additional component in the rule. For example, a decision maker may be moderately impressed by candidate 1 and gives an overall rating of 3. If we would give all components, including this rating, equal weights, we obtain *S* = 4 + 5 + 3 + 3 + 3 = 18. In general, this procedure does not result in better decisions compared to the procedures described above, but it also does not result in reduced accuracy, unless the holistic rating would receive a high weight (Sawyer, 1966). The advantage is that it increases the sense of control and autonomy of decision makers (e.g., Nolan and Highhouse, 2014).

### Method 4: Holistic Synthesis

Here, we start using a rule such as discussed in method 1 or 2, but after the scores have been calculated mechanically, the decision maker is allowed to combine the mechanical prediction with all other information holistically and may change the final rating accordingly. This enhances the feeling of autonomy. However, through the application of a rule resulting in a total score, decision makers obtain “anchors” that guide their judgments (Dietvorst et al., 2018). For example, let us assume that we first apply equal weights, and then the decision maker decides to add two points to the score of candidate 1 because of a skill that was perceived as useful but not explicitly assessed in the procedure. We hypothesize that this method probably yields more acceptance and higher use-intentions from decision makers compared to the “purer” mechanical procedures described previously, but lower accuracy as compared to pure mechanical rules without the possibility to overrule the results.

Finally, when a large pool of candidates is available, Kuncel (2008) proposed to first select candidates mechanically, and then select the final candidates holistically. This should lead to reasonably good results, because after the first mechanical hurdle, it is likely that most candidates are good candidates. Therefore, allowing the final hurdle to be holistic would not affect the decision quality that much.

## Commonly Raised Objections and Often Overlooked Advantages

### More Information Is Not Always Better

Many test and exam manuals warn against the over-interpretation of test scores. For example, in a document about the interpretation of Law School Admission Test (LSAT) scores (see the Law School Admission Council, 2014), the authors emphasize that “The LSAT is just one source of information that should be considered when evaluating an applicant.” Including more information may indeed be better, but only when that information increases the predictive validity and thus yields more accurate judgments and better decisions. What is often overlooked is that more information does not always lead to better prediction. When information is judged holistically, adding information with suboptimal validity, such as information from an unstructured interview, may even reduce validity, because it dilutes valid information obtained from, for example, grades or tests (Dana et al., 2013; Kausel et al., 2016). In addition, reduced validity as a result of adding less valid information to valid information can also occur when suboptimal weights are used, as we discussed above (Sackett et al., 2017). Therefore, it is advised to remove information that has small predictive validity and would be given a small weight from assessment procedures altogether (Wainer, 1978).

### Reliability of Scores and Ratings

A question that arises in discussions about mechanical judgment is how to take uncertainty and unreliability into account. For example, The Standards for Educational and Psychological testing (2014, p. 1) mentions that the standard error of measurment (SEM) should always be reported, and the Law School Admission Council (2014) states that the SEM should be considered when comparing LSAT scores between candidates.

Admittedly, scores and ratings are not perfectly reliable, and neither are the resulting total scores. For example, if we have two candidates for one position whose “suitability scores” are close together and whose confidence intervals overlap, what should we do? When making decisions, it is often inevitable that there will be persons with similar scores. In those cases, considering candidates whose confidence intervals overlap as similar is *not* an option because of the logical inconsistencies that follow from this approach (see Campion et al., 2001). Treating scores as interchangeable within a confidence interval will result in the absurd situation that we cannot distinguish any score within the whole range of test scores because “there are large numbers of scores below the bottom of the interval that are not statistically significantly different from most of the scores that are in that [confidence interval]” (Campion et al., 2001, p. 159).

Thus, how should we decide which candidate to select? Admittedly, the predictions about future performance based on the information we collect are probabilistic. However, the decisions we make based on probabilistic information usually are not; an applicant is either hired or rejected. Nevertheless, the best option is still to pick the candidates with the highest scores (possibly obtained across different predictors), and to ignore information about reliability or stability of scores in the part of the process in which decisions are made based on the information that was collected. Remember that a positive relationship between the measurements that are used to select a candidate and job performance ensures that higher scores will – on average – result in higher job performance.

Reliability should, of course, be considered when choosing what predictors to include in the decision-making process. Wainer (2005, p. 112) provided a very pragmatic argument to ignore stability of test scores when discussing admissions based on School Admission Test scores:

*“I certainly believe that the stability of scores* (…*) is important, but in most cases neither the applicant nor the schools receiving the scores care a lot about this information (other than the understanding that such scores are stable enough for their intended use). College admission tests are, to a very large extent, a contest. The focus is on who did best on a particular day. Olympic gold medals are not given out to the best athlete determined by averaging a large number of performances over a year or two.”*

Although one may argue that “applicants and schools should care,” taking uncertainty into account when binary decisions have to be made, such as accepting or rejecting a candidate, is practically not possible.

### Predicting Multiple Outcomes

In some cases, we may not want to predict one outcome, but multiple outcomes that are not strongly related to each other, such as task performance and turnover (Rubenstein et al., 2018), or diversity and adverse impact reduction. Indeed, predicting different outcomes may require different information and different weights (e.g., Darr and Catano, 2016). However, this does not mean that mechanical rules could not be used in these cases. We could use a form such as in Figure 1 for each outcome, and average the results across candidates or set cutoff values for each, or we can use both techniques. For example, as mentioned in the introduction with respect to admission testing, some colleges may want to take “hardship” and “service to the community” into account. We hope we have made clear that they can do so without having to use a holistic procedure. They can simply score such variables and then add that information to other variables they want to consider in a transparent way^3^. Another issue is the assumption that higher scores and ratings translate to higher job performance. However, if moderate scores are desirable, it is possible to give higher scores for moderate levels of a traits or skill in mechanical procedures, if that would be warranted.

### Transparency

An important advantage of mechanical decision-making is that decision makers can be completely transparent about how they reach a decision. Explicit and transparent rules to select candidates, and collecting feedback about the quality of selected candidates allow for evaluations and improvements of the procedure. How can we improve decision-making in personnel selection and college admission procedures if we do not know exactly how candidates have been selected? Transparent procedures allow for evaluation and improvement, but they also make the flaws and errors of judgments and decisions visible. That requires modesty in terms of promises made by decision makers in practice and the acceptance of errors from stakeholders about whom decisions are made.

### Limitations of Mechanical Judgments

To apply mechanical decision-making, as Dawes and Corrigan (1974, p. 105) discussed, “the whole trick is to decide what variables to look at and then to know how to add.” As we discussed above, this implies that we need valid predictors and that these predictors should sometimes be rescaled. Although this is certainly a surmountable obstacle, it calls for more human effort than combining information from different predictors in the mind, or based on an informal discussion. Perhaps the biggest limitation in practice is that decision makers do not like to apply mechanical decision-making because it weakens basic human needs such as autonomy, competence, and relatedness (Nolan and Highhouse, 2014). Therefore, we need more studies that investigate under which conditions practitioners do accept mechanical decision-making. Furthermore, it is important that the performance of the mechanical procedure remains under human supervision, which may sometimes be difficult because as Kahneman and Klein (2009, p. 524) discussed, “human operators become more passive and less vigilant when algorithms are in charge.”

## Improving Decision-Making in Practice

We have provided some guidelines on how mechanical rules can be implemented, taking common practical constraints into account, such as lack of (or small samples of) data. The methods we proposed for combining information are fast and frugal (Dana and Davis-Stober, 2016). For example, we combined scores on an ability test into different categories because we could not standardize these scores in a different way. As a result, we lost information. Therefore, when practitioners do have access to large databases, more sophisticated methods such as regression analysis, relative weights analysis, or dominance analysis are preferred over the procedures we described. However, we hope that with this paper, we have offered some guidelines on using mechanical judgment and decision-making in practice.

Of course, we understand that many hurdles need to be taken before mechanical judgment is used ubiquitously. This paper focused on making the information on the superiority of mechanical judgment over holistic judgment accessible for practitioners for everyday decision-making. Next steps will have to focus on attitude changes and increasing the ease of mechanical decision-making in practice. A first step is having convenient tools to place all different information on the same score scale, and working with decision makers in a co-production model to make mechanical judgment acceptable and accessible for everyone.

## Author Contributions

RM wrote the first version and other authors contributed by changing and adapting the text.

## Conflict of Interest

The authors declare that the research was conducted in the absence of any commercial or financial relationships that could be construed as a potential conflict of interest.

## Footnotes

1 We chose these assessments for this example because they are often used (e.g., Morris et al., 2015). However, these assessments are not (always) the most suitable assessments; this may depend on the position.

2 This approach is suboptimal compared to using standard scores (with a mean of zero and an SD of 1; see Bobko et al., 2007), and it reduces the variance of the cognitive ability test scores, which reduces predictive validity.

3 In some countries, this is not allowed for all variables associated with adverse impact reduction (see Bollinger, 2003).
